# Brief communication: The cohort of women prescribed HIV PrEP at the Veterans Health Administration

**DOI:** 10.1186/s12981-024-00670-z

**Published:** 2024-11-01

**Authors:** Shimrit Keddem, Kaitlyn Broderick, Puja Van Epps, Christopher B. Roberts, Sumedha Chhatre, Lauren A. Beste

**Affiliations:** 1 Corporal Michael J. Crescenz Veterans Affairs (VA) Center for Health Equity, Research & Promotion (CHERP), 4100 Chester Ave, Suite 203, Philadelphia, PA 19104 USA; 2 Department of Family Medicine & Community Health, Perelman School of Medicine, University of Pennsylvania, Philadelphia, PA USA; 3 Division of Infectious Diseases, Department of Medicine, Perelman School of Medicine, University of Pennsylvania, Philadelphia, PA USA; 4 Division of Infectious Diseases, VA Northeast Ohio Healthcare System, Louis Stokes Cleveland VA Medical Center, Cleveland, OH USA; 5 Department of Medicine, Division of Infectious Diseases and HIV Medicine, Case Western Reserve University School of Medicine, Cleveland, OH USA; 6 Department of Psychiatry, Perelman School of Medicine, University of Pennsylvania, Philadelphia, PA USA; 7 Division of General Internal Medicine, Department of Medicine, University of Washington School of Medicine, Seattle, WA USA; 8 General Medicine Service, VA Puget Sound Health Care System, Seattle, WA USA

**Keywords:** HIV pre-exposure prophylaxis (PrEP), Women Veterans, HIV prevention, Veterans Health Administration

## Abstract

The goal of this study was to describe the cohort of women prescribed PrEP at the Veterans Health Administration. We used a cross-sectional study of electronic health record data. We used descriptive statistics and calculated estimated average percent change by year of prescription. A total of 417 women were prescribed PrEP over the study period. The most substantial change over time in PrEP prescribing occurred among women aged 18–24, in Other race group, and in the Western US. Though PrEP prescribing increased since its approval, more research is needed to identify barriers and expand PrEP access for women Veterans.

## Introduction

HIV remains a persistent health concern in the United States, with over 36,000 new diagnoses in 2021 [1]. HIV Pre-exposure Prophylaxis (PrEP), recommended for persons at increased risk for HIV, is an evidence based practice that could help end the HIV epidemic. The Veterans Health Administration (VHA) is the largest single provider of HIV care in the US, but PrEP uptake among VHA patients has remained modest [2], especially among birth sex females, mirroring the general US population. This is especially concerning because women are more vulnerable to HIV infection than men since receptive anal or vaginal sex is riskier than insertive sex [3, 4], and given persisting societal gender inequalities that limit women’s autonomy [5, 6]. Moreover, there are considerable disparities in PrEP uptake, especially among Black and Hispanic women, whose rates of infection are drastically higher as compared with their white counterparts [7]. Missed opportunities to prevent HIV infection among women also have multigenerational consequences, given that HIV PrEP is a highly effective tool for preventing HIV infection and mother-to-infant transmission among women who are trying to conceive, pregnant, or breastfeeding [8]. As compared with non-Veterans, prevalence of military sexual trauma [9], intimate partner violence [10, 11] and mental health diagnoses [12, 13] may increase risk of HIV infection among women Veterans. In this study, we used VHA administrative data to describe the cohort of women Veterans prescribed PrEP at the VHA since its approval in 2012.

## Methods

This retrospective, cross-sectional study was approved by the Corporal Michael J. Crescenz VA Medical Center and granted a waiver of informed consent. The sample, extracted from the VHA’s Corporate Data Warehouse (CDW), included birth sex females enrolled in VHA care between January 1, 2012, and June 30, 2022, who were prescribed tenofovir disoproxil fumarate and emtricitabine (TDF/FTC) or tenofovir alafenamide and emtricitabine (TAF/FTC) for ≥ 30 days. To describe the demographics of the sample, we included age, race, and ethnicity. Homelessness, military sexual trauma, and intimate partner violence were based on the most recent screener in the medical record. Gonorrhea and chlamydia test results were obtained based on laboratory diagnostic tests performed in the 30 days before and 30 days after the start of the PrEP prescription. Mental health conditions from the medical record include alcohol use disorder, anxiety disorder, bipolar disorder, post-traumatic stress disorder (PTSD), schizophrenia and depressive disorders. US census region was based on patients’ home address. In addition, we examined all appointments in the 30 days prior to the PrEP prescription using stop codes in the electronic health record. We used descriptive statistics and calculated estimated average percent change (EAPC) by year of prescription by demographic characteristics using Poisson regression in Stata 17.0.

## Results

PrEP initiation increased gradually over the study years, with most patients receiving initial prescriptions between January 1, 2020 and June 30, 2022. Among the cohort of 417 women, 54% were aged 18–39, 33% were aged 40–54, and 13% were aged 55 years or older (Table 1). In terms of racial demographics, 53% identified as White, 40% as Black, and 7% as other. Hispanic women constituted 12% of the sample. 11% had experienced homelessness. Nearly half of the cohort (48%) experienced military sexual trauma. 15% were in combat service. Less than 2% of the sample had a positive gonorrhea or chlamydia test in the 30 days prior to starting their PrEP prescription. Regionally, in the Western US, 7.4 per 100,000 women were prescribed PrEP as compared with 4.9 per 100,000 in the Northeast and 5.1 per 100,000 in the Midwest. 75% of the sample had a primary care appointment and 65.5% had an infectious disease specialty appointment in the 30 days prior to their PrEP prescription. The most substantial change over time in PrEP prescribing occurred among women aged 18–24, in the ‘Other’ race group, among Hispanic women, and in the Western US (Table 2).

**Table 1:** Patient & provider characteristics among women Veterans receiving PrEP

Patient Characteristics	*n*	Per 100,000*	%
Age			
18–39	224	3.1	54.4
40–54	135	1.9	32.8
55+	53	0.7	12.9
Race			
White	219	3.0	53.2
Black	163	2.2	39.6
Other	29	0.4	7
Hispanic ethnicity	49	0.7	11.9
Homeless	44	0.6	10.7
Military Sexual Trauma	197	2.7	47.8
Intimate Partner Violence	27	0.4	6.6
Sexually transmitted infection			
Gonorrhea (30 day before PrEP initiation)	7	0.1	1.7
Chlamydia (30 days before PrEP initiation)	8	0.1	1.9
Gonorrhea (30 days after PrEP initiation)	1	< 0.1	0.5
Chlamydia (30 days after PrEP initiation)	2	< 0.1	0.2
Alcohol Use Disorder	113	1.6	27.4
Anxiety Disorders	325	4.5	78.9
Bipolar Disorder	142	1.9	34.5
PTSD	277	3.8	67.2
Schizophrenia	31	0.4	7.5
Depressive Disorders	350	4.8	85
Combat Service	60	0.8	14.6
U.S. Region**			
Northeast	35	4.9	8.4
Midwest	61	5.1	14.6
South	202	5.5	48.4
West	119	7.4	28.5
Appointment type in the last 30 days			
Primary Care	313	4.3	75.1
Infectious Disease	273	3.7	65.5
Mental Health	212	2.9	50.8
Diagnostic Imaging	73	1.0	17.5
Emergency	47	0.6	11.3
Prescriber Characteristics			
Advanced Practice Provider	92	1.3	22.1
Clinical Pharmacist	83	1.1	19.9
Physician	240	3.3	57.6
Other/unknown	2	0.0	0.5

*Rates per 100,000 were calculated based on total women Veteran population from 2012–2021

**US region rates per 100,000 were calculated based on regional populations of women Veterans from 2012–2021

**Table 2:** Count of HIV PrEP prescription among Women veterans by Year with EAPC

	2012		2013		2014		2015		2016		2017		2018		2019		2020		2021		EAPC	95% CI	
Race	**No.**	**%**	**No.**	**%**	**No.**	**%**	**No.**	**%**	**No.**	**%**	**No.**	**%**	**No.**	**%**	**No.**	**%**	**No.**	**%**	**No.**	**%**			
Black	1	(50)	1	(100)	1	(33)	5	(45)	16	(62)	13	(36)	23	(44)	19	(28)	29	(39)	31	(40)	35.4	26.1 -	45.4
Other	0	(0)	0	(0)	1	(33)	1	(9)	1	(4)	2	(6)	2	(4)	6	(9)	5	(7)	7	(9)	45.4	21.0 -	74.7
White	1	(50)	0	(0)	1	(33)	5	(45)	9	(35)	21	(58)	27	(52)	44	(64)	40	(54)	39	(51)	42.5	33.5 -	52.2
Age																							
18–24	0	(0)	0	0	0	(0)	0	(0)	1	(4)	2	(5)	1	(2)	3	(4)	4	(5)	4	(5)	60.2	21.1 -	111.8
25–34	0	(0)	0	0	1	(33)	3	(27)	7	(27)	10	(27)	14	(26)	21	(30)	26	(35)	27	(35)	48.4	35.5 -	62.7
35–44	0	(0)	1	100	0	(0)	3	(27)	6	(23)	8	(22)	22	(42)	21	(30)	22	(30)	27	(35)	45.9	33.5 -	59.4
45–54	0	(0)	0	0	0	(0)	3	(27)	8	(31)	12	(32)	10	(19)	12	(17)	11	(15)	14	(18)	34.6	21.5 -	49.2
> 55	2	(100)	0	0	2	(67)	2	(18)	4	(15)	5	(14)	6	(11)	12	(17)	11	(15)	5	(6)	25.5	12.3 -	40.3
Census Region																							
Midwest	0	(0)	0	0	0	(0)	1	(9)	4	(15)	7	(19)	9	(17)	7	(10)	8	(11)	15	(19)	43.6	26.5 -	62.9
Northeast	0	(0)	0	0	0	(0)	0	(0)	1	(4)	5	(14)	6	(11)	1	(1)	10	(14)	7	(9)	49.7	25.8 -	78.1
South	0	(0)	1	100	3	(100)	9	(82)	16	(62)	15	(41)	22	(42)	41	(59)	34	(46)	31	(40)	34.5	26.2 -	43.3
West	2	(100)	0	0	0	(0)	1	(9)	5	(19)	10	(27)	16	(30)	20	(29)	22	(30)	24	(31)	44.5	31.9 -	58.2
Ethnicity																							
Hispanic	0	(0)	0	0	0	(0)	1	(9)	1	(4)	3	(8)	5	(10)	12	(18)	11	(15)	9	(13)	53.2	31.6 -	78.3
Non-Hispanic	2	(100)	1	100	3	(100)	10	(91)	25	(96)	33	(92)	47	(90)	56	(82)	62	(85)	63	(88)	37.1	30.6 -	44.0
Total	2		1		3		11		26		37		53		69		74		77				

## Discussion

Although the number of women Veterans prescribed PrEP increased between 2012 and 2021, this cohort included only 417 women. Considering more than 1,300 women Veterans tested positive for chlamydia in 2019 alone, this study indicates PrEP is not reaching eligible women at the VHA. These findings parallel national patterns in PrEP use in the general population as well as those described for Veterans at large within the VHA [14, 15]. In this sample, 48% of women had a history of military sexual trauma, a higher proportion than documented rates among women Veterans [16]. This indicates that this sample of women is a vulnerable group given that military sexual trauma is tied to a host of mental health disorders including suicidal ideation, disordered eating, and substance use disorders [9]. By race, Black women Veterans saw the smallest increase in PrEP prescribing over time, which parallels disparities in non-Veterans. More efforts are needed to close the equity gap for women Veterans at highest risk of HIV. Lastly, very few women in this sample tested positive for gonorrhea or chlamydia in the 30 days prior to their PrEP prescription suggesting that a majority of the prescriptions were not related to STI infection. This is not surprising given that discussion and initiation of PrEP at the VHA are uncommon even following healthcare encounters for STIs [17].

Most prescribers were physicians, overwhelmingly infectious diseases providers, which differs from estimates from general and commercially insured populations, where primary care physicians prescribe most PrEP [14]. This finding merits further exploration but is potentially related to local requirements restricting PrEP prescribing to specialty care. Individuals at risk for acquiring HIV are more likely to seek and initiate PrEP care through their primary care provider (PCP), who are in an ideal position to screen for PrEP eligibility [18, 19]. Patterns of healthcare utilization in this sample in the 30 days prior to a PrEP prescription suggest that women first saw a PCP and were then referred to infectious disease for their PrEP care. Training PCPs and removing specialty prescribing restrictions will be important in lowering barriers to PrEP. One fifth of the patients in the study were prescribed PrEP by clinical pharmacists, and although data is lacking to compare to the general U.S., this suggests that Clinical Pharmacist Practitioners, who possess a scope of practice within the VHA which includes medication prescriptive authority and serving as a direct care provider, are an important tool to enhance PrEP uptake among women veterans. The prescribing patterns in our study suggest geographic disparities, with women living in the Western US having higher rates as compared with Midwest and Northeast. More research is needed to identify barriers and strategies to overcome them for PrEP service implementation among women Veterans.

## Data Availability

No datasets were generated or analysed during the current study.

## Abbreviations

CDW: Corporate Data Warehouse
EAPC: Estimated average percent change
PCP: Primary care provider
PrEP: HIV Pre-Exposure Prophylaxis
PTSD: Post-traumatic stress disorder
TAF/FTC: Tenofovir alafenamide and emtricitabine
TDF/FTC: Tenofovir disoproxil fumarate and emtricitabine
VHA: US Veterans’ Health Administration
VA: US Department of Veterans’ Affairs
